# Deconstructing Oral Surgical and Orthodontic Considerations in Complex Cases: A Case Report on Two Patients With Multiple Impacted Teeth

**DOI:** 10.1155/crid/6984311

**Published:** 2026-09-07

**Authors:** Brendon Anderson, Heidi Shen, Juanna Xie, David Cabeceiras, Nalton Ferraro

**Affiliations:** ^1^ Harvard School of Dental Medicine, Boston, Massachusetts, USA, harvard.edu; ^2^ Department of Orofacial Sciences, University of California San Francisco School of Dentistry, San Francisco, California, USA, ucsf.edu; ^3^ Department of Orthodontics, Tufts University School of Dental Medicine, Boston, Massachusetts, USA, tufts.edu; ^4^ Department of Oral and Maxillofacial Surgery, Harvard School of Dental Medicine, Boston, Massachusetts, USA, harvard.edu

**Keywords:** case report, multiple impacted teeth, nonsyndromic, orthodontic–surgical management, supernumerary teeth

## Abstract

Management of multiple impacted teeth in nonsyndromic adolescents can be challenging, particularly when impactions involve several quadrants. This case report describes two adolescent patients with extensive impactions affecting both permanent and supernumerary teeth, with an emphasis on the clinical reasoning that guided treatment decisions. A quadrant‐by‐quadrant approach was used to determine when teeth were extracted, surgically exposed, or retained based on eruption potential, root development, and surgical access. Although the anatomical complexity of the cases was similar, treatment strategies differed according to individualized risk–benefit considerations. These cases highlight the value of structured clinical judgment in managing complex impactions without relying on rigid treatment algorithms.

## 1. Introduction

Tooth impaction, defined as the failure of a tooth to erupt into its expected position within the dental arch, is a common dental condition that affects around 0.8%–3.6% of the general population [1, 2]. The most frequently impacted teeth are mandibular third molars, followed by maxillary third molars and maxillary canines [1]. Etiology is multifactorial, involving systemic, local, and genetic factors. Common contributors include failure of deciduous tooth resorption, abnormal eruption paths, tooth‐size arch length discrepancies, supernumerary teeth, cysts, trauma, and hereditary anomalies such as malposed tooth germs or cleidocranial dysostosis [1, 3, 4]. Although single tooth impactions are extremely common, with third molar impactions affecting over 90% of individuals, cases involving synchronous impactions of molars, bicuspids, canines, and supernumerary teeth in multiple quadrants are much rarer [5]. Although complex tooth impactions are encountered in clinical practice, guidance on systematic decision‐making in patients with synchronous, multiquadrant impactions involving permanent and supernumerary teeth remains limited. These presentations require individualized, staged planning that integrates anatomical complexity, orthodontic objectives, and surgical risk.

Prior case reports have described individual treatment decisions in patients with multiple impacted teeth. However, most focus on outcomes rather than the clinical reasoning behind those decisions [6–10]. In complex impaction cases, clinicians are often required to balance competing priorities, including occlusal symmetry, tooth‐size arch length discrepancies, eruption potential, surgical accessibility, and long‐term contingency planning. The absence of a structured framework for navigating these clinical decisions can make management unpredictable, particularly in young, growing patients.

This case report presents the diagnosis and management of multiple impacted teeth in two adolescent patients using a quadrant‐by‐quadrant framework that emphasizes clinical reasoning rather than prescriptive algorithms. Treatment decisions are presented in tabular form and correlated with diagnostic imaging to illustrate how anatomical, orthodontic, and surgical considerations were integrated into each stage. These cases are intended to demonstrate an adaptable approach to complex impactions rather than defining a single standard of care.

## 2. Case Presentation

### 2.1. Case #1: 10‐Year‐Old Nonsyndromic Patient

Case #1 involves a 10‐year‐old nonsyndromic female patient who presented for surgical management of extensive multiquadrant impactions and supernumerary teeth. Management focused on preserving eruption potential where feasible while maintaining arch symmetry and minimizing surgical morbidity. Decisions were made on a quadrant‐by‐quadrant basis, allowing treatment sequencing as eruption patterns became clearer over time.

**Figure 1:**
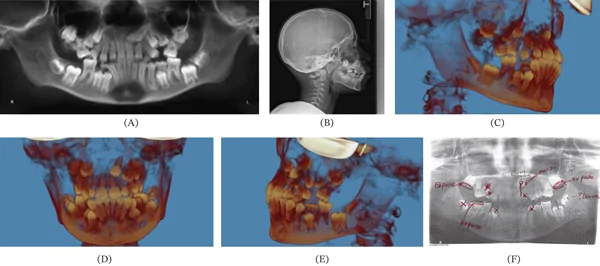
(A) Case #1 preliminary panoramic radiograph. (B) Case #1 preliminary lateral cephalogram. (C–E) Case #1 3D dentoalveolar reconstruction to determine tooth position. (F) Case #1 handwritten planning on panoramic radiograph.

The panoramic radiograph and lateral cephalogram (Figure 1A,B) are available to help follow the tabular tooth‐by‐tooth decisions in Table 1. A 3D CT reconstruction is included, which is moderately helpful (Figure 1C–E). A panoramic radiograph with handwritten planning is also provided and often serves as a reasonable planning exercise (Figure 1F).

**Table 1:** Treatment decisions for Case #1 (10‐year‐old nonsyndromic patient).

Quadrant	Tooth #	Outcome	Reason and comments
Upper right	1	Congenitally missing	
	2	Significant removal of coronal soft tissue	Crown exposure to aid in eruption
	3	Retained	
	A	Exfoliated	
	4	Retained	
	5	Extracted	Maintain balance with the upper left quadrant
	6	Retained	
	7	Retained	
	8	Retained	

Upper left	9	Retained despite root resorption	
	10	Extracted, significant root resorption	Allow guided eruption of #11
	11	Exposed and bracketed, subtotal enucleation of cyst	Note the dentigerous cyst surrounding the crown of #11
	12	Retained	
	13	Retained	
	14	Retained	
	15	Significant removal of coronal soft tissue	Crown exposure to aid in eruption
	16	Congenitally missing	

Lower left	17	Retained	Uncertainty regarding #18 eruption potential
	18	Exposed and bracketed	
	19	Retained	
	20	Retained	
	21	Extracted	
	22	Retained	
	23	Retained	
	24	Retained	

Lower right	25	Retained	
	26	Retained	
	27	Retained	
	28	Extracted	Facilitate eruption of #29
	29	Retained	Anticipating eruption
	30	Retained	
	31	Retained	
	32	Extracted	Impacted

#### 2.1.1. Posttreatment

**Figure 2:**
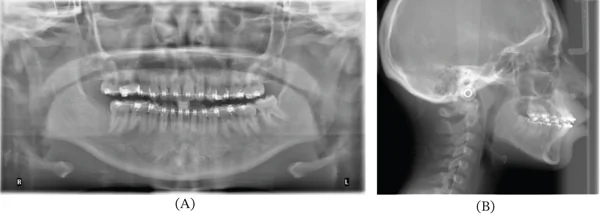
(A) Case #1 posttreatment panoramic radiograph. (B) Case #1 posttreatment lateral cephalogram.

Treatment progressed over multiple staged surgical and orthodontic interventions spanning early adolescence, with reassessment at each phase guiding subsequent decisions. These posttreatment images represent the lateral cephalogram and panoramic radiograph (Figure 2A,B) obtained at completion of active orthodontic therapy.

Tooth #17 erupted but remains crowded with slight supraeruption. It was intentionally retained as a contingency tooth due to uncertainty regarding the orthodontic feasibility of guiding the deeply positioned #18 into the arch. Ultimately, #18 erupted into the arch very favorably. Teeth #2 and #15, which were initially covered by a greater amount of soft tissue, also erupted successfully. No adverse or unanticipated events occurred during treatment.

#### 2.1.2. Long‐Term Follow‐Up

**Figure 3:**
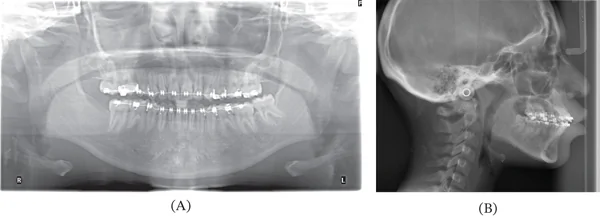
(A) Case #1 panoramic radiograph long‐term follow‐up. (B) Case #1 lateral cephalogram long‐term follow‐up.

A follow‐up visit was completed at the age of 16. Active orthodontic treatment for this patient involved the placement of a rapid palatal expander at age 10 and removal at age 11. Next, fixed appliance therapy was performed until debonding at age 15. The patient was evaluated for long‐term follow‐up 44 weeks after debonding (Figure 3A,B). Functional tooth counts at completion of treatment were symmetric across quadrants (upper right: 6; upper left: 6; lower left: 7; and lower right: 6). Tooth #17, previously retained as a contingency plan in case #18 failed to erupt, was noted to have an asymptomatic operculum on the distal aspect of the crown. Because #18 was effectively guided into occlusion, #17 no longer served a functional purpose, and removal was planned. The patient and her mother were informed of this recommendation and consented to extraction. A minor diastema was noted between Teeth #11 and #12 by the patient′s mother. However, continued orthodontic treatment was not recommended at this time due to limited functional or esthetic gain. The patient reported a positive recollection of the surgical procedure, recalling the need for some postoperative analgesics but no lasting negative impressions of the operation or postoperative recovery. Both the patient and her mother expressed satisfaction with the esthetics, function, and transformation from initial presentation.

### 2.2. Case #2: 13‐Year‐Old Nonsyndromic Patient

**Figure 4:**
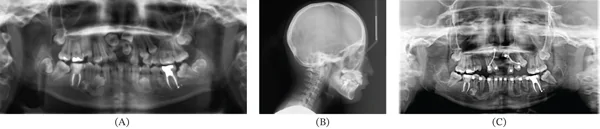
(A) Case #2 preliminary panoramic radiograph. (B) Case #2 preliminary lateral cephalogram. (C) Case #2 panoramic radiograph during treatment.

Case #2 involves a 13‐year‐old nonsyndromic female patient who presented for surgical management of multiple supernumerary and impacted teeth, including two mesiodens. As in Case #1, management prioritized preserving structurally sound teeth when surgical access posed a significant risk. Decisions to retain or extract teeth were guided not only by radiographic position but also by anticipated surgical morbidity and orthodontic utility. Additionally, a panoramic radiograph and lateral cephalogram are available to follow the tabular tooth‐by‐tooth decisions in Table 2 that were made (Figure 4A,B).

**Table 2:** Treatment decisions for Case #2 (13‐year‐old nonsyndromic patient).

Quadrant	Tooth #	Outcome	Reason and comments
Upper right	1	Retained	While removal was possible, the tooth was positioned superiorly within the alveolar arch, making surgical access unfavorable. Plan: future removal
	2	Retained	
	3	Retained	
	4	Retained	
	5	Extracted	To allow adequate room for Tooth #s 4, 6, and 7
	6	Retained	
	7	Retained	
	8	Bracketed and repositioned	Deeply impacted and horizontal, imaging is crucial to determine structural viability

Upper left	Mesiodens x2	Localized and surgically removed	
	9	Bracketed and repositioned	Deeply impacted and horizontal
	10	Retained	
	11	Retained	Unerupted but well‐positioned
	12	Retained	Unerupted but well‐positioned
	13	Extracted	
	J	Exfoliated	
	14	Retained	
	15	Retained	
	16	Retained	While removal was possible, the tooth was positioned superiorly within the alveolar arch, making surgical access unfavorable. Plan: future removal

Lower left	17	Extracted	To facilitate the uprighting of #18
	18	Retained and luxated	
	19	Retained	
	20	Retained	
	20’	Extracted	Supernumerary impacted lower premolars can be technically difficult to remove
	21	Retained	
	21’	Extracted	
	22	Retained	
	23	Retained	
	24	Retained	

Lower right	25	Congenitally missing	Unusual for this tooth to be missing
	26	Retained	
	27	Retained	
	28	Retained	
	28’	Extracted	
	29	Retained	
	29’	Retained	Complexity and risk of removal
	30	Retained	
	31	Retained and luxated	
	32	Extracted	To facilitate the uprighting of #31

The midtreatment panoramic radiograph (Figure 4C) demonstrates improved positioning of Teeth #18 and #31. Brackets and chains are visible on Teeth #8 and #9. The supernumerary impacted lateral premolar was deliberately retained, as removal posed a high risk of damage to adjacent roots and neurovascular structures without a clear functional benefit.

#### 2.2.1. Posttreatment

**Figure 5:**
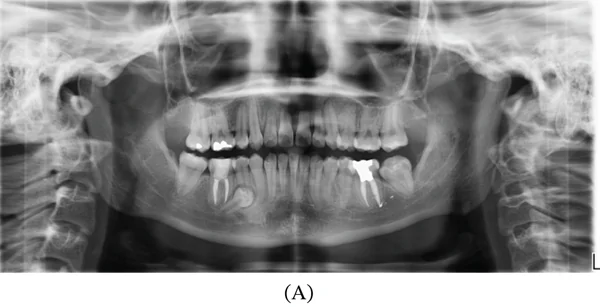
(A) Case #2 posttreatment panoramic radiograph.

Management of Case #2 involved staged surgical and orthodontic interventions performed over early adolescence, with periodic reassessment guiding decisions regarding extraction, exposure, and retention. These posttreatment images demonstrate the panoramic radiographic findings at the completion of active orthodontic therapy (Figure 5A). No adverse or unanticipated events occurred during treatment.

#### 2.2.2. Long‐Term Follow‐Up

**Figure 6:**
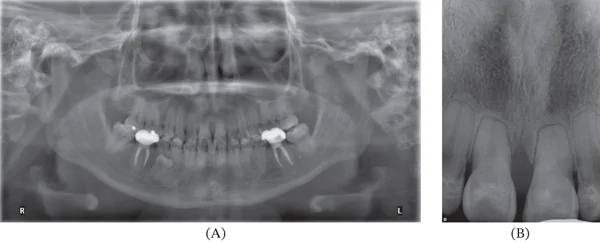
(A) Case #2 panoramic radiograph long‐term follow‐up. (B) Case #2 periapical radiograph long‐term follow‐up.

At a follow‐up visit at the age of 21, following a total active orthodontic treatment period of 60 months, the patient′s occlusion remained stable and favorable. Follow‐up imaging was taken at this appointment and paired with the clinical examination, confirming surgical and orthodontic stability. Functional tooth counts at completion of treatment were symmetric across quadrants (upper right: 6; upper left: 6; lower left: 7; and lower right: 7). A dermal graft had been placed over the anterior segment (Teeth #7–#10) to address the soft tissue deficiency. Teeth #8 and #9 required ongoing periodontal monitoring, with blunted root morphology and Grade 1 mobility; the patient was maintained in an Essix retainer and advised to avoid incising with these teeth. #8 and #9 root resorption is likely associated with the distance traveled through orthodontically guided eruption (Figure 6B). Keratinized tissue at the site remained favorable. Should mobility worsen, anterior splinting or future implant therapy may be indicated. Tooth #18 was found to have deep caries and flagged for extraction. Caries burden was assessed at follow‐up and determined to be high in this patient. The etiology of #18′s decay was not attributed to orthodontic care or the performed surgical procedure. The area surrounding retained #29 ^′^ demonstrated no radiographic evidence of external root resorption. On clinical examination, no buccal or lingual cortical bulge was appreciated overlying the retained supernumerary premolar (#29 ^′^), suggesting an intimate anatomic relationship with the root of the adjacent #29. Given this close proximity, the risk of iatrogenic damage to #29 during removal of #29 ^′^ was judged to outweigh any functional benefit of extraction, supporting the original risk‐based decision to leave it in place. The patient reported no recollection of symptoms following the surgical procedure and expressed satisfaction with the surgical and orthodontic outcome. Figure 6A,B reflects imaging taken at the follow‐up appointment.

## 3. Discussion

Institutional review board approval was not required for this case report, as it involved retrospective review of deidentified clinical data. Written informed consent for publication of this case report and accompanying images was obtained from the patients and/or their legal guardians. Identifying information has been removed to protect patient confidentiality.

In patients with extensive dental impactions, surgical and orthodontic decision‐making must extend beyond radiographic position alone. In these cases, five interrelated factors guided treatment planning: dental symmetry, anterior–posterior compensation, lateral occlusal relationships, dental crowding, and surgical accessibility. Rather than applying uniform extraction criteria, each tooth was evaluated for its functional contribution, eruption trajectory, surgical risk, and long‐term orthodontic value. This approach allowed the treatment plan to remain adaptable as eruption patterns evolved.

For both patients, a standardized imaging protocol was utilized to characterize the position, orientation, and surgical accessibility of impacted and supernumerary teeth. This protocol consisted of a panoramic radiograph and lateral cephalogram for initial visualization and localization, supplemented by three‐dimensional cone beam computed tomography (CBCT) as necessary to picture more intimate spatial relationships. Consistent with the American Academy of Oral and Maxillofacial Radiology guidance, CBCT was used on a case‐by‐case basis, being individually justified in situations where two‐dimensional imaging was insufficient to localize impacted or supernumerary teeth or characterize their proximity to adjacent teeth or neurovascular structures, while also maintaining the principle of reducing radiation exposure, especially in pediatric populations [11]. While no formal radiographic classification was applied, this combination of two‐ and three‐dimensional imaging reflects standard practice for the assessment of complex tooth impactions.

**Figure 7:**
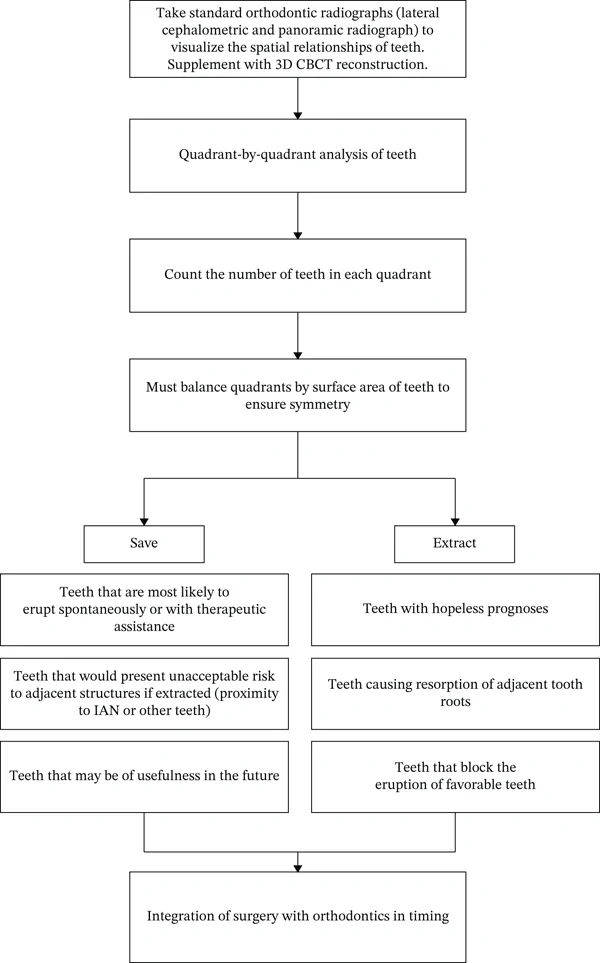
Decision tree framework.

Once imaging was obtained, the dentition was separated into quadrants for evaluation. Within each quadrant, the number of erupted, impacted, and supernumerary teeth was recorded, and each tooth was assigned a long‐term restorative potential. Restorative potential was based on crown structure, position, accessibility, ease of removal, and relative functional importance in its respective quadrant. Teeth judged structurally or periodontally hopeless, or actively impeding the eruption of a more favorably positioned tooth, were prioritized for extraction. Teeth with sound structure but questionable or poor surgical access, where removal presented excessive risk of damaging adjacent neurovascular structures or adjacent roots, were retained. In selected cases, teeth were maintained to preserve future restorative options. This approach was guided by the orthodontic principle that symmetry between quadrants, meaning a comparable amount of retained tooth structure, rather than identical tooth types, supports stable occlusion and midline balance. Figure 7 illustrates the decision‐making protocol applied across both cases.

This framework does not eliminate the role of clinical judgment, nor does it produce identical outcomes across patients with similar presentations, as demonstrated through Cases #1 and #2. Rather, it provides a consistent and reproducible sequence of imaging, quadrant separation, tooth‐by‐tooth prognosis, and risk‐based extraction or retention decisions for organizing clinical judgment in complex patient cases.

Existing literature on the management of impacted teeth emphasizes assessment of eruption potential, root development, and surgical accessibility when determining treatment strategies. Previous case reports of nonsyndromic patients with multiple impacted or supernumerary teeth primarily focus on diagnostic findings, with management decisions guided by associated pathology, eruption disturbance, or surgical risk [6–10]. While these reports underscore the importance of early diagnosis and multidisciplinary evaluation, they generally apply these considerations at the level of individual teeth rather than within a structured, longitudinal framework spanning multiple quadrants. The present cases build on these observations by providing a structured, quadrant‐based approach that makes underlying clinical reasoning explicit and allows for flexibility as eruption patterns and orthodontic feasibility evolve over time.

A common line of thought in managing complex impactions is whether a more aggressive extraction strategy would simplify treatment. In both cases presented, teeth were selectively retained when extractions posed disproportionate surgical risk, or when retained teeth could serve as contingency options should primary orthodontic objectives fail. A more aggressive extraction‐only protocol, removing all impacted and supernumerary teeth regardless of position or prognosis, would have simplified surgical planning. However, such an approach would have sacrificed teeth with reasonable eruption potential or failed to consider teeth whose extraction may exhibit more risk than benefit. This case report does not propose the quadrant‐based approach as a defined standard of care. Rather, it presents one rational method that can be easily reproduced in clinical practice, with adaptability and personalization to the individual patient.

In Case #1, emphasis was placed on facilitating eruption and maintaining occlusal balance and symmetry in the arch. Several decisions demonstrate how eruption potential and preservation of healthy root structure guided management. Tooth #10 was extracted due to significant root resorption and to aid in guiding the eruption of Tooth #11, which was exposed and subsequently bracketed after partial enucleation of the cyst. Similarly, extraction of #28 was performed to facilitate eruption of #29, reflecting a space‐creation strategy aimed at preserving teeth with favorable eruption potential. Selective retention was also used as a deliberate contingency strategy. Tooth #17 was retained due to uncertainty regarding the eruption potential of #18, preserving a functional option should eruption fail. Of note, #18 eventually was bracketed and brought into the arch once a successful eruption was more certain. Some may have considered an alternate approach, extracting #17 and relying on #18. However, the retention of #17 presented limited risk while providing time to make a more accurate assessment of eruption potential. Notably, Tooth #9 was retained despite root resorption because the root structure remained stable, underscoring that retention decisions were based on individualized, clinical assessments rather than radiographic findings alone. A quadrant‐based framework was used to evaluate each tooth in relation to adjacent teeth and overall arch balance, allowing eruption‐guided decisions to be coordinated within the broader treatment plan rather than made in isolation.

In Case #2, treatment strategy heavily relied on surgical feasibility and risk to adjacent structures, despite similarly extensive, multiquadrant supernumeraries. Several teeth were retained specifically because removal posed unfavorable access or disproportionate surgical risk. For example, Maxillary Third Molars #1 and #16 were retained due to their superior position within the arch, making surgical access unfavorable. Likewise, Supernumerary Tooth #29 ^′^ was retained due to complexity and risk of removal. Eruption potential should not be the sole factor determining treatment options, as extraction may present substantial risk to the adjacent hard and soft tissues. Teeth #8 and #9, both deeply impacted and horizontally positioned, were surgically exposed, bracketed, and orthodontically repositioned following imaging assessment to confirm viability. Elsewhere, selective extractions were performed to facilitate uprighting rather than eruption, including removal of #17 to assist uprighting of #18 and removal of #32 to facilitate uprighting of #31, which was luxated and retained. Applying the same quadrant‐based framework helped prioritize risk mitigation across affected quadrants, ensuring that retention, extraction, or deferral decisions reflected both local anatomy and their impact on adjacent teeth.

Although both cases involved nonsyndromic adolescents with extensive, multiquadrant impactions, similar clinical scenarios prompted different management priorities. In Case #1, extractions and surgical exposures were frequently used to create space and actively guide eruption when orthodontic benefit outweighed risk, as demonstrated by management of #10/#11 and #28/29. In Case #2, comparable anatomical challenges led to more conservative strategies when surgical morbidity was considered. Several teeth were ultimately retained due to unfavorable access or elevated risk, including #1, #16, and #29 ^′^. Importantly, similar strategies such as selective retention were employed in both cases: ensuring the presence of a functional molar in Case #1 and minimizing surgical risk in Case #2.

Together, these cases illustrate the complexities inherent in managing multiquadrant impactions and the limitation of applying a one‐size‐fits‐all management strategy. This case report illustrates an adaptable framework that remains systematic in its implementation. Grounded in the quadrant‐based approach, this method of analysis confers an individualized risk‐benefit analysis to each tooth. By outlining our rationale behind each decision, we hope to offer a model that clinicians can apply to their complex cases.

This case report is limited by its small sample size and retrospective nature, which preclude a control or comparison group. Treatment decisions were influenced by individual anatomical considerations and interdisciplinary expertise, which may limit generalizability and external validity in other clinical settings; although treatment rationale was presented transparently in Tables 1 and 2, clinician‐driven selection bias cannot be fully excluded. Additionally, although follow‐up data, including patient‐reported outcomes, were obtained beyond completion of active orthodontic therapy for both patients, these data were collected retrospectively at follow‐up rather than prospectively at the time of treatment, and long‐term periodontal outcomes for all retained teeth remain to be fully established. Nonetheless, these cases illustrate a reproducible framework for clinical reasoning in situations where standardized guidelines are lacking.

## 4. Conclusion

The successful management of multiple impacted teeth requires a flexible, quadrant‐by‐quadrant strategy that considers each tooth′s anatomical position, eruption potential, and functional value. These cases demonstrate that treatment planning must extend beyond radiographic interpretation to include a deeper analysis of occlusion, growth potential, and surgical risk. While no single algorithm fits every case, structured clinical reasoning provides a reliable foundation for navigating the unpredictability of complex impactions. Together, these cases illustrate how structured clinical reasoning can guide flexible, patient‐specific management of complex impactions without relying on a single prescriptive pathway.

## Funding

No funding was received for this manuscript.

## Conflicts of Interest

The authors declare no conflicts of interest.

## Data Availability

The data that support the findings of this study are available from the corresponding author upon reasonable request.

## References

[1] Chu F. C. S. , Li T. K. L. , Lui V. K. B. , Newsome P. R. H. , Chow R. L. K. , and Cheung L. K. , Prevalence of Impacted Teeth and Associated Pathologies: A Radiographic Study of the Hong Kong Chinese Population, Hong Kong Medical Journal. (2003) 9, no. 3, 158–163, 12777649. 12777649

[2] Hattab F. N. , Rawashdeh M. A. , and Fahmy M. S. , Impaction Status of Third Molars in Jordanian Students, Oral Surgery, Oral Medicine, Oral Pathology, Oral Radiology, and Endodontology. (1995) 79, no. 1, 24–29, 10.1016/s1079-2104(05)80068-x, 7614155. 7614155

[3] Becker A. , Orthodontic Treatment of Impacted Teeth, 2012, 1st edition, John Wiley & Sons, 10.1002/9781118709641.

[4] Power S. M. and Short M. B. , An Investigation Into the Response of Palatally Displaced Canines to the Removal of Deciduous Canines and an Assessment of Factors Contributing to Favourable Eruption, British Journal of Orthodontics. (1993) 20, no. 3, 215–223, 10.1179/bjo.20.3.215, 8399054. 8399054

[5] American Association of Oral and Maxillofacial Surgeons , Impacted Wisdom Teeth, 2025, AAOMS, https://myoms.org/what-we-do/wisdom-teeth-management/impacted-wisdom-teeth/. 9667187

[6] Schubert M. , Proff P. , and Kirschneck C. , Successful Treatment of Multiple Bilateral Impactions: A Case Report, Head & Face Medicine. (2016) 12, no. 1, 10.1186/s13005-016-0122-0, 27457490. PMC4960832 27457490

[7] Ajith S. D. , Shetty S. , Hussain H. , Nagaraj T. , and Srinath M. , Management of Multiple Impacted Teeth: A Case Report and Review, Journal of international Oral health: JIOH. (2014) 6, no. 3, 93–98, 25083041. 25083041 PMC4109248

[8] Ahammed H. , Seema T. , Deepak C. , and Ashish J. , Surgical Management of Impacted Supernumerary Tooth: A Case Series, International Journal of Clinical Pediatric Dentistry. (2021) 14, no. 5, 726–729, 10.5005/jp-journals-10005-2008, 34934291. 34934291 PMC8645632

[9] Wimalarathna A. K. , Non-Syndromic Multiple Unerupted Supernumerary Teeth : A Case Report, Journal of the Postgraduate Institute of Medicine. (2016) 3, 1–5, 10.4038/jpgim.8083.

[10] Thumati P. , David C. M. , and Tiwari R. , Nonsyndromic Multiple Supernumerary Teeth: A Case Report of 11 Supernumerary Teeth, Journal of Indian Academy of Oral Medicine and Radiology. (2012) 24, no. 5, 296–299, 10.5005/jp-journals-10011-1317.

[11] American Academy of Oral and Maxillofacial Radiology , Clinical Recommendations Regarding Use of Cone Beam Computed Tomography in Orthodontics. [corrected]. Position Statement by the American Academy of Oral and Maxillofacial Radiology, Oral Surgery Oral Medicine Oral Pathology Oral Radiology. (2013) 116, no. 2, 238–257, 10.1016/j.oooo.2013.06.002, 23849378. 23849378

